# Prevalence, genetic diversity and antiretroviral drugs resistance-associated mutations among untreated HIV-1-infected pregnant women in Gabon, central Africa

**DOI:** 10.1186/1471-2334-12-64

**Published:** 2012-03-20

**Authors:** Mélanie Caron, Sonia Etenna Lekana-Douki, Maria Makuwa, Guy-Patrick Obiang-Ndong, Olivia Biba, Dieudonné Nkoghé, Mirdad Kazanji

**Affiliations:** 1 Unité de Rétrovirologie, Centre International de Recherches Médicales de Franceville, Franceville BP 769, Gabon; 2 Programme National de lutte contre le SIDA, Libreville BP 50, Gabon; 3 Ministère de la Santé, Libreville BP 5879, Gabon; 4 Institut Pasteur de Bangui, Réseau International des Instituts Pasteur, Bangui BP 923, Central African Republic

**Keywords:** HIV-1, Prevalence, Genetic diversity, Resistance to antiretroviral drugs, Untreated pregnant women, Gabon, Central Africa

## Abstract

**Background:**

In Africa, the wide genetic diversity of HIV has resulted in emergence of new strains, rapid spread of this virus in sub-Saharan populations and therefore spread of the HIV epidemic throughout the continent.

**Methods:**

To determine the prevalence of antibodies to HIV among a high-risk population in Gabon, 1098 and 2916 samples were collected from pregnant women in 2005 and 2008, respectively. HIV genotypes were evaluated in 107 HIV-1-positive samples to determine the circulating subtypes of strains and their resistance to antiretroviral drugs (ARVs).

**Results:**

The seroprevalences were 6.3% in 2005 and 6.0% in 2008. The main subtype was recombinant CRF02_AG (46.7%), followed by the subtypes A (19.6%), G (10.3%), F (4.7%), H (1.9%) and D (0.9%) and the complex recombinants CRF06_cpx (1.9%) and CRF11_cpx (1.9%); 12.1% of subtypes could not be characterized. Analysis of ARVs resistance to the protease and reverse transcriptase coding regions showed mutations associated with extensive subtype polymorphism. In the present study, the HIV strains showed reduced susceptibility to ARVs (2.8%), particularly to protease inhibitors (1.9%) and nucleoside reverse transcriptase inhibitors (0.9%).

**Conclusions:**

The evolving genetic diversity of HIV calls for continuous monitoring of its molecular epidemiology in Gabon and in other central African countries.

## Background

Human immunodeficiency virus type 1 (HIV-1) is the main sexually transmitted infectious agent in the world, affecting approximately 33 million people with 2.7 million new infections each year [1]. Diseases associated with HIV infection cause 2.0 million of deaths annually, and about 19 million people have already died from this cause. Most HIV-1 infections are observed in Africa, where the widest genetic diversity has been described [2]. Previous studies showed that the HIV-1 pandemic originated in central Africa, where populations have been in close contact with pure subtypes, unique and complex recombinant forms and many unclassified strains [3-6].

Studies in the past decade showed increasing genetic diversity of HIV-1 in Gabon, central Africa [7-10]. In 2000, Makuwa *et al. *demonstrated a predominance of subtype A (49%) in the general population, with a number of unclassified HIV-1 strains (13%) [8]. Two years later, the CRF02_AG (26%) and complex recombinant MAL-like lineage (19%) were found to be increasing [10]. A recent study conducted in our laboratory showed a predominance of CRF02_AG (57%) among manganese miners [7]. However, a high proportion of subtypes (30%) were found discordant, indicating a complex genetic composition of these circulating HIV-1 strains in Gabon. A further study from our laboratory confirmed stable circulation of CRF02_AG (63%) among migrant populations, and for the first time, demonstrated the presence of the complex recombinant form CRF11_cpx [9].

The anti-HIV prevalence infection in the general Gabonese population is difficult to determine. Although HIV prevention programs and social conditions have improved, only a few patients in the main cities have free access to treatment in medical structures with the capacity to treat sexually transmitted diseases [11]. Nevertheless, some studies have been conducted on the resistance of HIV-1 to antiretroviral drugs (ARVs). A study in Libreville, the capital of Gabon, showed emerging resistance to nucleoside reverse transcriptase inhibitors (NRTIs) during treatment for HIV-1 infection [12]. Two other studies, conducted among untreated population, identified only mutations associated with subtype polymorphism and did not address susceptibility to ARVs [7,9]. Data on HIV drug resistance-associated mutations among untreated and treated population in resource-limited settings such as Gabon are either scarce or out of date.

To limit the propagation of HIV, native or transmitted ARVs-resistant strains must be detected by continuous monitoring of HIV-infected population at greatest risk. In the absence of an effective vaccine against HIV, this information is needed before new introduction of ARVs in Africa. In order to evaluate the level of resistance to ARVs in Gabon, we determined the anti-HIV prevalence among pregnant women in the main cities to characterize the circulating subtypes and ARVs resistance-associated mutations.

## Methods

### Area and population studied

**Figure 1:**
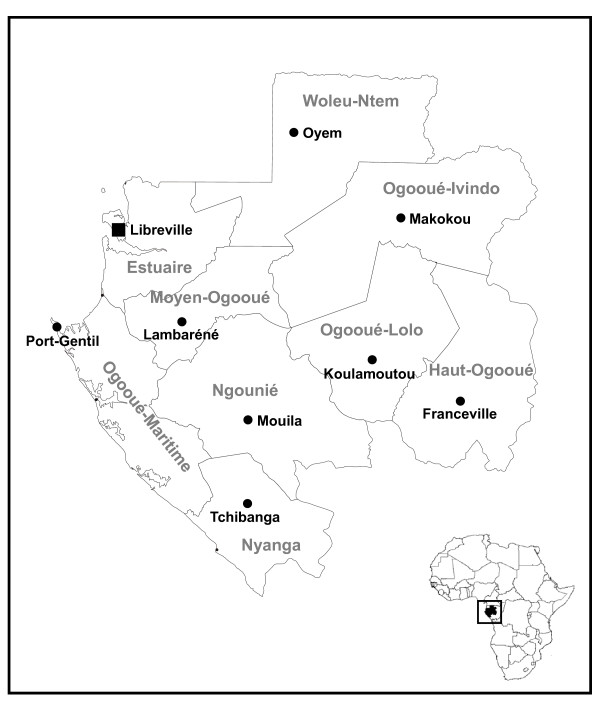
**Map of Gabon, central Africa, with provinces and main cities**. In grey, provinces; square, capital; circles, main cities.

Gabon occupies 26 7667 km^2 ^in the Gulf of Guinea on the Equator, with tropical forest covering three quarters of the territory. The population is around 1.5 million, 73% of whom live in urban areas. Gabon is divided into nine provinces, in which the main cities are: Libreville (Estuaire), the capital of Gabon, in the northwest; Port-Gentil (Ogooué-Maritime), the main harbor and economic capital, in the west; Lambaréné (Moyen-Ogooué) in the centre west; Oyem (Woleu-Ntem) in the north; Makokou (Ogooué-Ivindo) in the northeast; Franceville (Haut-Ogooué) in the southeast; Koulamoutou (Ogooué-Lolo) in the centre east; Mouila (Ngounié) in the centre and Tchibanga (Nyanga) in the south of the country (Figure 1).

In 2005 and 2008, the retrovirology laboratory at the International Centre for Medical Research of Franceville (CIRMF) and the National Program to Fight Against AIDS in Libreville conducted epidemiologic surveys of pregnant women at their first antenatal examination or first postnatal medical consultation, according to the guidelines of the World Health Organization. The study reported here was conducted in the main cities of the nine provinces, thus representing the general population of pregnant women in Gabon. The survey was conducted by a multidisciplinary team, including physicians from the Gabonese Ministry of Health. Anonymous venous blood samples were collected by locally trained health staff. Before testing, fully informed consent was obtained from each woman and when women were younger than 18 years, informed consent was obtained from their parents. The samples were anonymous, but age and geographic origin were retained. The study obtained ethical clearance from the Gabonese public health authorities and from the Gabonese scientific and ethical committees.

A total of 1098 samples were collected in 2005 and 2916 samples in 2008. The mean age of the women was 24.5 ± 6.3 years (range, 14-44 years) in 2005 and 24.0 ± 6.4 years (range, 14-47 years) in 2008. To determine the circulating subtypes and HIV-resistance to ARVs, samples were taken from 107 untreated HIV-1-infected pregnant women aged 26.6 ± 6.4 years (range, 15-40 years) and included for molecular and phylogenetic analysis (data not shown). The women was selected on the basis of a high viral load (≥ 4.0 log_10 _copies/mL; mean, 5.7 ± 6.3 log_10 _copies/mL; range, 4.0-7.3 log_10 _copies/mL) in order to obviate failure of *pol *gene amplification.

### Serological analysis

Plasma samples were separated from venous blood and tested for the presence of antibodies to HIV-1 or HIV-2 by ELISA (Genscreen^® ^HIV-1/2 version 2, Biorad, Marne la Coquette, France) and by a rapid test (Determine™ HIV-1/2, Abbott, Chicago, Illinois, USA), according to the manufacturers' instructions. Antibody pattern was confirmed by western blot (New LAV Blot I & II, BioRad, Marne la Coquette, France).

### Molecular and phylogenetic analysis

RNA was extracted from plasma samples with a QIAamp^® ^Viral RNA Mini Kit (Qiagen, Courtaboeuf, France), and viral plasma RNA from HIV-positive samples was used to quantify the viral load (Generic HIV Charge Viral, Biocentric, Bandol, France). In order to determine the HIV subtypes and resistance-associated mutations in protease (P) and reverse transcriptase (RT) coding regions, *pol *gene targets of the main ARVs, were amplified by the French National Agency for AIDS Research consensus polymerase chain reaction (PCR) technique (AC-11 Resistance Group, http://www.hivfrenchresistance.org).

HIV genotype was determined by local alignment of partial *pol *sequences with the HIV Drug Resistance Database of Stanford University http://dbpartners.stanford.edu/RegaSubtyping/ and the HIV Database of the National Institutes of Health http://www.hiv.lanl.gov/content/sequence/BASIC_BLAST/basic_blast.html. To confirm the assignment of HIV subtypes, reference sequences obtained from the Los Alamos Sequence Database and each Gabonese sequence were aligned with the ClustalX program [13]. Manual corrections were performed with the editor program of the MEGA package, and phylogenetic relations were reconstructed by the neighbor-joining method [14,15]. Bootstrapping was performed with 1000 replicates.

Genotypic resistance to ARVs was defined according to the 2011 Stanford Resistance Surveillance list of critical mutations http://hivdb.stanford.edu, the latest versions of the algorithms of the French National Agency for AIDS Research and the International AIDS Society http://www.iasusa.org. We confirmed our results by determining transmitted ARVs resistance-associated mutations in untreated women, as recommended by Bennett et al. [16].

The newly sequenced HIV-1 strains are available under GenBank accession numbers HQ541872-HQ541944 and HQ541945-HQ541956.

### Statistical analysis

Analyses were performed with Epi-Info (version 6.04 dfr, ENSP-Epiconcept-InUS, 2001). A *p *value < 0.05 was considered statistically significant. Results are expressed as percentages (with 95% confidence intervals, CIs) and odds ratios (ORs, with 95% CIs). Spearman correlation coefficients were used to assess correlations between age groups and anti-HIV prevalences. In order to avoid selection bias in the molecular and phylogenetic analysis, the Kruskal-Wallis test was used to compare continuous variables among more than two groups; for age groups by viral load and by city. No difference was found (data not shown).

## Results

### Anti-HIV-1 prevalence

Antibodies to HIV were found in 69 of 1098 pregnant women tested in 2005 (6.3%; 95% CI, 4.9-7.7) and 174 of 2916 in 2008 (6.0%; 95% CI, 5.7-6.3) (Table 1). No significant difference between 2005 and 2008 was observed by age group or by province (data not shown). During both years of the study, seroprevalences increased by age group, with the highest among women aged ≥ 36 years and the lowest among those aged ≤ 15 years. ELISA and western blotting indicated some HIV-2 (n = 3, data not shown); no HIV-1 group O or N was found in our population. HIV-2-infected women were therefore excluded from the molecular studies.

**Table 1:** Prevalence of HIV by age among untreated pregnant women in Gabon in 2005 and 2008

Age group (years)	2005			2008		
		
	HIV+/tested	% [95% CI]	OR [95% CI]	HIV+/tested	% [95% CI]	OR [95% CI]
≤ 15	1/41	2.4 [1.5-3.3]	0.4 [0.1-2.7]	3/106	2.8 [2.2-3.4]	0.5 [0.1-1.4]
16-25	35/619	5.7 [4.3-7.1]	0.8 [0.5-1.3]	78/1732	4.5 [3.7-5.3]	0.5 [0.4-0.7]
26-35	26/336	7.7 [6.1-9.3]	1.4 [0.9-2.3]	74/899	8.2 [7.2-9.2]	1.7 [1.3-2.4]
≥ 36	7/72	9.7 [7.9-11.5]	1.7 [0.7-3.8]	19/179	10.6 [9.5-11.7]	2.0 [1.2-3.3]
Total	69/1098	6.3 [4.9-7.7]	-	174/2916	6.0 [5.7-6.3]-	

HIV+/tested, number of HIV-positive and number of samples tested; CI, confidence interval; OR, odds ratio

### HIV-1 subtypes and phylogenetic analysis

**Figure 2:**
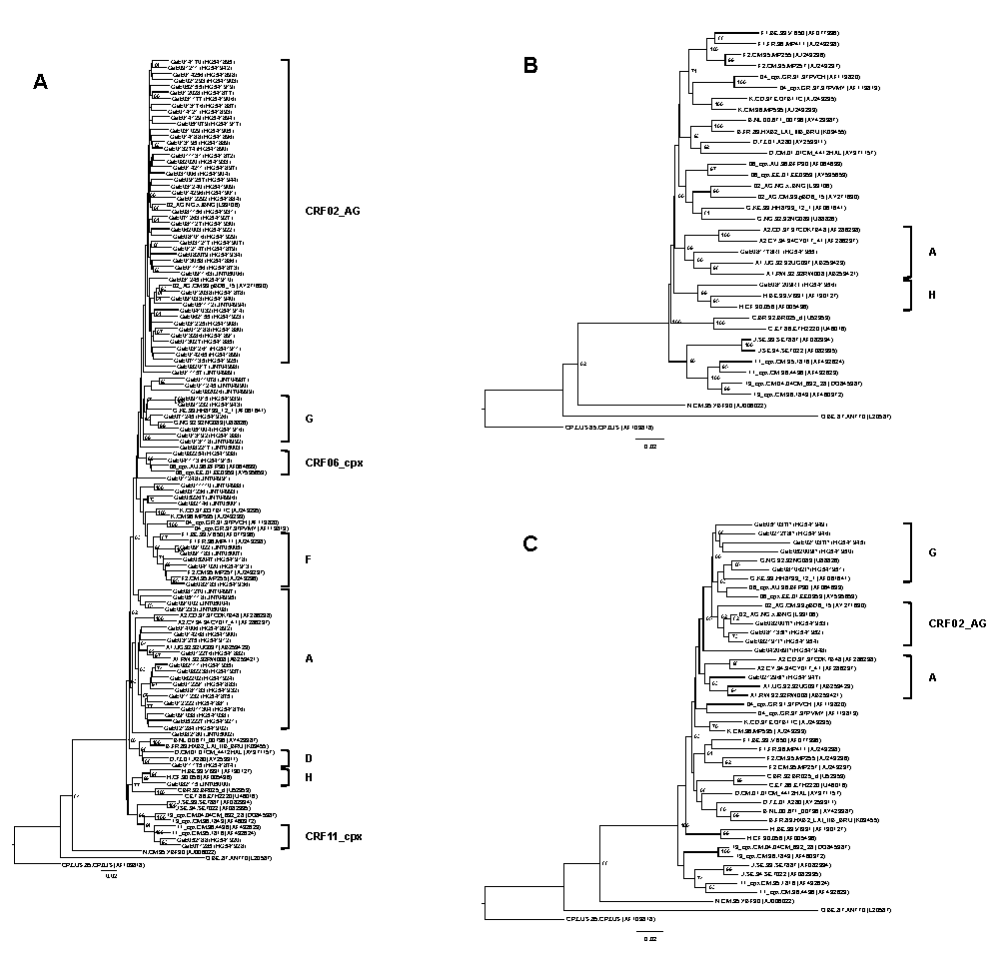
**Phylogenetic reconstructions for the assignment of HIV-1 subtypes, including the newly sequenced strain, in Gabon, central Africa**. (**A**) Phylogenetic tree of the partial *pol *gene constructed with a > 1000-bp fragment (n = 95; range, 1004-1073 bp); (**B**) phylogenetic tree of reverse transcriptase coding region (n = 2; 785 bp); (**C**) phylogenetic tree of protease coding region (n = 10; range, 467-479 bp). Dataset of sequences was compiled by combining reference sequences representative of groups M (each pure subtype and commonest CRFs), N and O. The CPZ.US.85.CPZUS (AF103818) strain was used as outgroup to root the trees. The neighbor-joining method was used for the partial *pol *gene, reverse transcriptase and protease coding regions. Confidence levels were estimated for 1000 replicates, and only bootstrapping values higher than 60% were considered significant.

To evaluate the circulating HIV-1 subtypes, a > 1000 base-pair (bp) fragment (n = 95; range, 1004-1073 bp) of the *pol *gene (P and RT coding regions) was sequenced and phylogenetically analyzed (Figure 2A). The long fragment of the *pol *gene could not be obtained for 12 of the 107 HIV-1 samples, and analyzed either the RT (n = 2; 785 bp) or the P (n = 10; range, 467-479 bp) coding region (Figure 2B and 2C).

Phylogenetic analysis of the *pol *gene showed wide genetic diversity (Figure 2A, B and 2C). The genotypes distribution is shown in Table 2. The main subtype identified was CRF02_AG (n = 50, 46.7%), followed by subtypes A (n = 21, 19.6%), G (n = 11, 10.3%), F (n = 5, 4.7%), H (n = 2, 1.9%) and D (n = 1, 0.9%). Complex recombinant forms were also found, consisting of CRF06_cpx and CRF11_cpx (n = 2, 1.9%). Thirteen subtypes could not be characterized (12.1%).

**Table 2:** Minor and major resistance-associated mutations to protease inhibitors (PIs), nucleoside reverse transcriptase inhibitors (NRTIs) and non-NRTIs of HIV-1 strains from untreated pregnant women in Gabon

Subtype	n/N	PIs	NRTIs	non-NRTIs
A	2/21	L10V	-	V106I, V179I
	1/21	-	L210W*	E138A
	2/21	-	-	V179I
	16/21	-/ND	-/ND	-/ND
D	1/1	-	-	V179I
F	3/5	L10V	-	-
	2/5	-	-	-
G	1/11	L10I	ND	ND
	1/11	L10I, V11I	ND	ND
	1/11	L10I, V11I, M46I*	ND	ND
	1/11	-	V118I	-
	1/11	-	-	A98S
	2/11	-	-	V179E
	4/11	-	-/ND	-/ND
H	2/2	-/ND	-	-
CRF02_AG	2/50	L10I	-	-
	2/50	L10V	-	-
	1/50	L10V, **L33F**	-	-
	1/50	L10V, T74S	-	-
	2/50	E35G	-	-
	1/50	M46I*	-	V90I
	1/50	-	T69S	-
	2/50	-	-	V90I
	1/50	-	-	V90I, V179I
	1/50	-	-	K101Q
	1/50	-	-	K103R
	2/50	-	-	V179I
	33/50	-	-/ND	-/ND
CRF06_cpx	2/2	-	-	-
CRF11_cpx	1/2	L10I	-	-
	1/2	-	-	-
Und	3/13	L10I	-	-
	1/13	L10I	-	V106I
	2/13	L10V	-/ND	-/ND
	1/13	L10V	-	V179I
	1/13	L10I, A71T	-	V179I
	1/13	-	A62V	-
	1/13	-	V118I	-
	3/13	-	-	-

n, number of HIV-1 strains with resistance-associated mutations to PIs, NRTIs and non-NRTIs; in boldface, major mutation; -, no mutation; ND, not done; *, major mutation according to the list of investigations on transmitted ARVs resistance-associated mutations in untreated individuals [16]

### ARVs resistance-associated mutations

Resistance to ARVs was genotyped in 107 HIV-1-positive samples. Data on mutations conferring resistance to protease inhibitors (PIs), nucleoside reverse transcriptase inhibitors (NRTIs) and non-NRTIs are summarized in Table 2. In all, 44/107 (41.1%) HIV-1 strains presented minor or major genotypic resistance to ARVs, of which 3/107 (2.8%) were major mutations for resistance to PIs (n = 2/107, 1.9%) and NRTIs (n = 1/107, 0.9%). No major mutation for resistance to non-NRTIs was found.

The most frequent mutations observed in the P coding region corresponded to subtype polymorphism (data not shown), while minor mutations, such as L10V/I, V11I, E35G, A71T and T74S, were associated with low-level resistance to some PIs (Table 2). L33F and M46I indicate major PI resistance. In the RT coding region, strong polymorphism was found (data not shown), with common mutations such as V90I, V106I and E138A (Table 2). A62V and T69S are minor mutations, which alone do not affect susceptibility to NRTIs, like K103R and V179I to non-NRTIs. L210W contributes to resistance to NRTIs, and K101Q causes low-level reduction in susceptibility to non-NRTIs. However, the only mutation known to be associated with resistance to NRTIs was V118I.

Six of the 44 (13.6%) HIV-1 strains had mutations for resistance to PIs and non-NRTIs, and one (2.3%) was resistant to both NRTIs and non-NRTIs. Five strains (11.4%) cumulated mutations to PIs, and three (6.8%) cumulated mutations to non-NRTIs (Table 2).

## Discussion

The prevalence of antibodies to HIV was 6.1%, with no significant difference between 2005 and 2008 or by age group. Although few data are available for Gabon, the prevalence of antibodies to HIV between 1986 and 1989 was about 2.0% in the general population of Libreville [17]. More recent studies on populations at risk in Gabon showed a seroprevalence of 4.5% among manganese miners and 7.5% in the general population living close to Cameroon [7,9].

Of note, migrant populations and pregnant women had higher seroprevalences than miners in Gabon; this finding might be due to the geographic location of sampling. The low seroprevalence among manganese miners might be due to containment of this semi-rural population, whereas migrant populations and pregnant women are more representative of the general urban population. Furthermore, the mining company educated workers about HIV transmission, perhaps resulting in a 'healthy worker effect'. Similar findings were made in Cameroon in 2004, with seroprevalences of 4.0% and 6.7% in rural and urban populations [18]. In the Central African Republic in 2006, a prevalence of 6.2% was found in Bangui and 7.9% for the rest of country, with a seroprevalence almost twice as high among women than men [19].

Our results show that widely genetically diverse HIV-1 strains are circulating among untreated pregnant women in Gabon, including pure subtypes (A, D, F, G, H), a unique recombinant form (CRF02_AG) and complex recombinant forms (CRF06_cpx, CRF11_cpx). At least eight viral lineages have been observed, confirming the results of previous studies in central Africa [7-10]. As a number of strains remained unclassified, genetic diversity in Gabon is probably even greater, with complex mosaic compositions. A wide genetic diversity of circulating strains was also reported in the Democratic Republic of the Congo and Cameroon [20,21]. We confirmed that CRF02_AG is still the most prevalent HIV-1 subtype throughout Gabon. This subtype was widespread in west Africa a few years ago and is now present in central Africa [22].

The geographic distribution of HIV subtypes is changing constantly, with the greatest genetic diversity of HIV-1 in sub-Saharan Africa [23]. A preliminary study in the Democratic Republic of the Congo determined pure subtypes of two HIV strains, and further investigations revealed a combination of several subtypes [24]. In Cameroon, unique recombinant forms have been found to be far more complex than previously described, indicating evolution of unique forms into complex combinations [25]. Recombinant forms were reported to have greater replicative capacity *in vitro *than the pure subtypes from which they derived [26]. In our study, the viral load of HIV-1 strains among untreated pregnant women was high, suggesting a potentially critical role of dual infections *in vivo*, as the attained HIV viral load could limit the frequency of inter-subtype recombination [27].

Vergne *et al. *reported resistance to ARVs among patients in Gabon followed up for HIV infection. No other studies in this country have addressed direct effects on the effectiveness of the most frequently used ARVs in HIV-1 treatment. As expected, high frequencies of polymorphism mutations were found in the P and RT coding regions, which do not affect the three classes of ARVs. Even in HIV-1 strains from untreated patients, mutations associated with susceptibility to PIs, NRTIs and non-NRTIs were observed. The L33F and M46I mutations reduce susceptibility to PIs, except for saquinavir/r; L210W confers low-level resistance to several NRTIs, except for lamivudine and emtricitabine; V118I induces low-level resistance to lamivudine and K101Q causes a low-level reduction in susceptibility to non-NRTIs. These findings confirm resistance to HIV drugs among untreated patients infected with non-B subtypes, even though these drugs were introduced in Gabon relatively recently.

Some ARVs resistance-associated mutations occur commonly without selective pressure at highly polymorphic positions; these polymorphic mutations should therefore not be considered in surveillance of transmitted resistance to ARVs [16], as their inclusion leads to inflated estimates. We found that 2.8% were major mutations for resistance to PIs (1.9%) and NRTIs (0.9%), providing little evidence for transmitted resistance to ARVs among untreated pregnant women in central Africa.

In the present study, we evaluated resistance in 107 samples obtained from strains in the active phase of viral replication, representing about the half of the HIV-positive women. As these samples contain strains from all regions of Gabon, they give an overall picture of natural ARVs resistance-associated mutations in this population. We cannot exclude the possibility that other ARVs resistance-associated mutations were present among strains from samples not evaluated here. Therefore, the true level of resistance might be underestimated, and epidemiological studies should be conducted in the general population each year to follow-up the circulating subtypes of the HIV strains and to evaluate the emergence of new ARVs resistance-associated mutations.

The emergence of strains with both minor and major mutations for one or several classes of ARVs poses a real problem, especially for the introduction of new ARVs or switch therapy for HIV-infected patients. Furthermore, the risk for resistance to ARVs treatment might be exacerbated by cumulated mutations. Although experts have dismissed the relevance of changes related to polymorphic mutations of non-B subtypes, low-to-moderate resistance to HIV drugs has been reported in neighboring central African countries in which high HIV diversity was found [28,29].

## Conclusions

A high prevalence of antibodies to HIV, broad genetic diversity and increased resistance to ARVs among untreated pregnant women in Gabon demonstrate that the epidemic features are now similar to those in other central African countries. Moreover, our findings highlight the potential consequences for molecular diagnosis and follow-up of HIV-infected patients. Without control of the HIV-1 load by ARVs treatment, viruses replicate actively, increasing polymorphism or accumulated minor mutations among these circulating strains. Thus, these mutations can promote the transmission of strains resistant to ARVs. Regular monitoring of the dynamics of HIV-1 in this country must therefore continue, and there is an urgent need to assess the impact of these mutations on the effectiveness of ARVs therapy.

## Competing interests

The authors declare that they have no competing interests.

## Authors' contributions

MC, SELD, MM carried out the serological and molecular studies. GPON, OB and DN contributed to the collection of sera and compiled the epidemiologic data. MC, DN and MK participated in the design of study, the statistical analysis and the drafting of manuscript. All authors read and approved the final version of the manuscript.

## Pre-publication history

The pre-publication history for this paper can be accessed here:

http://www.biomedcentral.com/1471-2334/12/64/prepub
